# Design and rationale of the ODYSSEY DM-DYSLIPIDEMIA trial: lipid-lowering efficacy and safety of alirocumab in individuals with type 2 diabetes and mixed dyslipidaemia at high cardiovascular risk

**DOI:** 10.1186/s12933-017-0552-4

**Published:** 2017-05-25

**Authors:** Dirk Müller-Wieland, Lawrence A. Leiter, Bertrand Cariou, Alexia Letierce, Helen M. Colhoun, Stefano Del Prato, Robert R. Henry, Francisco J. Tinahones, Lisa Aurand, Jaman Maroni, Kausik K. Ray, Maja Bujas-Bobanovic

**Affiliations:** 1 Department of Internal Medicine I, University Hospital Aachen, Pauwelsstr. 30, 52074 Aachen, Germany; 2 Li Ka Shing Knowledge Institute and Keenan Research Centre for Biomedical Science, St. Michael’s Hospital, University of Toronto, Toronto, ON Canada; 3 Institut du Thorax, CHU Nantes, Nantes, France; 4 Biostatistics and Programming Department, Sanofi, Chilly-Mazarin, France; 5 University of Edinburgh, Edinburgh, Scotland UK; 6 Department of Clinical and Experimental Medicine, University of Pisa, Pisa, Italy; 7 University of California San Diego School of Medicine, Center for Metabolic Research, Veterans Affairs, San Diego Healthcare System, San Diego, CA USA; 8 CIBERobn, Hospital Virgen de la Victoria, Málaga University, Málaga, Spain; 9 Sanofi, Bridgewater, NJ USA; 10 Regeneron Pharmaceuticals Inc., Tarrytown, NY USA; 11 Imperial Centre for Cardiovascular Disease Prevention, Department of Primary Care and Public Health, Imperial College, London, UK; 12 Sanofi, Paris, France

**Keywords:** Alirocumab, PCSK9, Diabetes, Mixed dyslipidaemia, Non-HDL-C, ODYSSEY

## Abstract

**Background:**

Type 2 diabetes mellitus (T2DM) is often associated with mixed dyslipidaemia, where non-high-density lipoprotein cholesterol (non-HDL-C) levels may more closely align with cardiovascular risk than low-density lipoprotein cholesterol (LDL-C). We describe the design and rationale of the ODYSSEY DM-DYSLIPIDEMIA study that assesses the efficacy and safety of alirocumab, a proprotein convertase subtilisin/kexin type 9 (PCSK9) inhibitor, versus lipid-lowering usual care in individuals with T2DM and mixed dyslipidaemia at high cardiovascular risk with non-HDL-C inadequately controlled despite maximally tolerated statin therapy. For the first time, atherogenic cholesterol-lowering with a PCSK9 inhibitor will be assessed with non-HDL-C as the primary endpoint with usual care as the comparator.

**Methods:**

DM-DYSLIPIDEMIA is a Phase 3b/4, randomised, open-label, parallel group, multinational study that planned to enrol 420 individuals. Main inclusion criteria were T2DM and mixed dyslipidaemia (non-HDL-C ≥100 mg/dl [≥2.59 mmol/l], and triglycerides ≥150 and <500 mg/dl [≥1.70 and <5.65 mmol/l]) with documented atherosclerotic cardiovascular disease or ≥1 additional cardiovascular risk factor. Participants were randomised (2:1) to alirocumab 75 mg every 2 weeks (Q2W) or lipid-lowering usual care on top of maximally tolerated statin (or no statin if intolerant). If randomised to usual care, investigators were able to add their pre-specified choice of one of the following to the patient’s current statin regimen: ezetimibe, fenofibrate, omega-3 fatty acids or nicotinic acid, in accordance with local standard-of-care. Alirocumab-treated individuals with non-HDL-C ≥100 mg/dl at week 8 will undergo a blinded dose increase to 150 mg Q2W at week 12. The primary efficacy endpoint is non-HDL-C change from baseline to week 24 with alirocumab versus usual care; other lipid levels (including LDL-C), glycaemia-related measures, safety and tolerability will also be assessed. Alirocumab will be compared to fenofibrate in a secondary analysis.

**Results:**

Recruitment completed with 413 individuals randomised in 14 countries worldwide. Results of this trial are expected in the second quarter of 2017.

**Conclusions:**

ODYSSEY DM-DYSLIPIDEMIA will provide information on the efficacy and safety of alirocumab versus lipid-lowering usual care in individuals with T2DM and mixed dyslipidaemia at high cardiovascular risk using non-HDL-C as the primary efficacy endpoint.

*Trial registration* NCT02642159 (registered December 24, 2015)

**Electronic supplementary material:**

The online version of this article (doi:10.1186/s12933-017-0552-4) contains supplementary material, which is available to authorized users.

## Background

Cardiovascular disease is a significant cause of morbidity and mortality in persons with type 2 diabetes mellitus (T2DM) [1]. Individuals with T2DM often have elevated levels of non-high-density lipoprotein cholesterol (non-HDL-C), triglycerides (TGs), and small, dense low-density lipoprotein (LDL), which further increase their cardiovascular risk. Mixed dyslipidaemia is commonly associated with insulin resistance, which accounts for increased hepatic production of very low-density lipoprotein (VLDL), enhanced intestinal production of chylomicrons, and reduced hepatic clearance of TG-rich lipoproteins (TRL) [2]. The management of mixed dyslipidaemia is a persistent challenge in clinical practice [3].

The relationship between LDL cholesterol (LDL-C) and cardiovascular risk is well established [4]. However, non-HDL-C, which can easily be calculated from a routine lipid panel by subtracting HDL-C from total cholesterol (TC) [5], is considered a better predictor of cardiovascular risk, particularly in individuals with T2DM and mixed dyslipidaemia [6–8]. Non-HDL-C has been proposed as a therapeutic target for mixed dyslipidaemia since it encompasses all circulating atherogenic cholesterol carried by apolipoprotein (Apo) B-containing lipoprotein particles, like TRL, including VLDL, intermediate-density lipoprotein (IDL), LDL and lipoprotein(a) [Lp(a)]. The National Lipid Association recommendations indicate non-HDL-C as a co-primary, but superior, treatment target with LDL-C [9]. Recent European guidelines [4, 10] recommend non-HDL-C as a practical alternative treatment target. However, the effect of novel lipid-lowering treatments on this parameter is still poorly studied.

Most guidelines/consensus statements suggest a non-HDL-C target of 30 mg/dl above target LDL-C levels. Therefore, for individuals with diabetes mellitus (DM) at very-high or high cardiovascular risk who have LDL-C goals of <70 or <100 mg/dl, respectively, the recommended non-HDL-C levels are <100 or <130 mg/dl [9–12].

Maximally tolerated statin therapy is commonly used to manage mixed dyslipidaemia, particularly with the goal of reducing LDL-C and non-HDL-C levels. Significant reductions in cardiovascular events have been observed in individuals with DM treated with statins [13]. However, real-world studies often report under-utilisation of statins and sub-optimal dosing in high-risk patients [14, 15]. Additional lipid-lowering therapies are used in conjunction with statins to address mixed dyslipidaemia, including ezetimibe, fibrates, nicotinic acid and omega-3 fatty acids. Although these additional therapies have not definitively demonstrated cardiovascular outcome benefits in several large randomised trials, in a post hoc subgroup analysis, fibrates have been associated with an apparent reduction in cardiovascular events in patients with mixed dyslipidaemia [16]. Fibrate therapy was most beneficial when TG levels were lowered, particularly in individuals with high baseline TG levels [16, 17]. Despite these treatment options, many individuals with DM continue to have persistent lipid abnormalities [18–20], and are therefore exposed to residual cardiovascular risk [21–23].

Alirocumab is a fully human monoclonal antibody that binds to and inhibits proprotein convertase subtilisin/kexin type 9 (PCSK9), a key regulator of plasma LDL-C metabolism. Alirocumab is approved in many countries, including the USA and across Europe, for the management of patients with hypercholesterolaemia on maximally tolerated statin therapy. As demonstrated in the ODYSSEY Phase 3 clinical studies, alirocumab reduces LDL-C levels by up to 60%, both alone and in combination with statin and other lipid-lowering therapies, in patients with dyslipidaemia [24]. In contrast to bococizumab (a murine-derived, humanised monoclonal antibody to PCSK9 that was recently discontinued from clinical development due to the attenuation of LDL-C lowering over time and higher rates of immunogenicity) [25, 26], LDL-C reductions on alirocumab did not decrease over time and very low rates of immunogenicity were observed. Such evidence suggests differences in immunogenicity potential for murine-derived antibodies humanised by in vitro artificial engineering, versus fully human antibodies that were produced with mice with genetically humanised immune systems [27].

In addition to LDL-C lowering, alirocumab significantly reduces non-HDL-C, Apo B and Lp(a), and trends for reduction in TGs, along with modest increases in HDL-C and Apo A-1, were also observed [24]. Data from a post hoc analysis of the ODYSSEY LONG TERM trial have been promising, with early indication of a reduced rate of cardiovascular events with alirocumab [28]. The benefit of alirocumab on cardiovascular morbidity and mortality will be assessed in the ongoing ODYSSEY OUTCOMES trial (NCT01663402), which will include over 18,000 patients randomised within 1 year post acute coronary syndrome, many of whom also have DM [29].

Sub-analyses of individual and pooled data from ODYSSEY Phase 3 studies showed a similar efficacy and safety profile in individuals receiving alirocumab with and without DM [30, 31], with no effect on glycaemic control [32]. In a pooled subgroup analysis of five Phase 3 trials of individuals with DM and mixed dyslipidaemia, alirocumab substantially improved LDL-C and non-HDL-C levels versus placebo, with similar results and safety profiles observed when compared with individuals without mixed dyslipidaemia [33]. While this analysis addresses an important clinical question, it remains unknown what is the best treatment strategy to manage lipid abnormalities and reduce cardiovascular burden in individuals with DM and mixed dyslipidaemia on maximally tolerated statin therapy. Furthermore, the efficacy and safety of alirocumab in this patient population has yet to be assessed in a dedicated study in the ODYSSEY programme, nor has any other clinical trial studying PCSK9 inhibitors utilised non-HDL-C as the primary endpoint.

Here, we describe the design and rationale for the ODYSSEY DM-DYSLIPIDEMIA study, which evaluates the superiority of alirocumab versus lipid-lowering usual care, utilising non-HDL-C as the primary efficacy endpoint, in individuals with T2DM and mixed dyslipidaemia at high cardiovascular risk with non-HDL-C not adequately controlled with maximally tolerated statin therapy. Usual care included optional addition of one of the following lipid-lowering therapies: ezetimibe, fenofibrate, omega-3 fatty acids or nicotinic acid. Findings from this head-to-head study of alirocumab versus lipid-lowering usual care will inform clinical decision-making beyond maximally tolerated statin therapy. In a secondary analysis, superiority of alirocumab versus fenofibrate on non-HDL-C (and all other efficacy endpoints measured in the primary analysis versus usual care) will also be assessed.

## Methods

### Study design

**Fig. 1:**
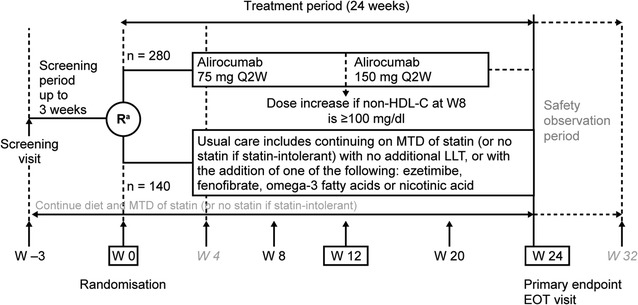
Study design. *EOT* end of treatment, *LLT* lipid-lowering therapy, *MTD* maximally tolerated dose, *non*-*HDL*-*C* non-high-density lipoprotein cholesterol, *Q2W* every 2 weeks, *R* randomisation, *W* week. ^a^First study drug administration. As a principle, randomisation should occur after signature of the informed consent form and just before the first dosing of the study drug (i.e. alirocumab or usual care). The randomisation day is always day 1. Randomisation was stratified by the investigator’s selection of usual care therapy prior to randomisation. Phone call visits are indicated in *italics*

ODYSSEY DM-DYSLIPIDEMIA (ClinicalTrials.gov identifier: NCT02642159) is a Phase 3b/4, randomised, open-label, parallel group, multi-centre, multinational clinical trial. The study evaluates the efficacy and safety of alirocumab versus lipid-lowering usual care in the reduction of non-HDL-C in individuals with T2DM and mixed dyslipidaemia at high cardiovascular risk with non-HDL-C not adequately controlled with maximally tolerated statin therapy (Fig. 1). The planned population for this study was 420 individuals. This trial began recruitment in March 2016 with main study results expected in the second quarter of 2017.

This clinical trial is being performed in accordance with the ethical principles outlined at the 18th World Medical Assembly (Helsinki, 1964) and all applicable amendments by the World Medical Assemblies and International Conference Harmonization Guidelines for Good Clinical Practice. Institutional review board or independent ethics committee approval of the protocols and informed consent forms from each study site (Additional file 1: Appendix S1) have been obtained, and written informed consent has been obtained from all study participants.

### Inclusion and exclusion criteria

Inclusion and exclusion criteria are shown in Table 1. A full list and details of inclusion and exclusion criteria can be found in Additional file 1: Appendix S2.

**Table 1:** Inclusion and exclusion criteria

Inclusion criteria
Aged ≥18 years or legal age of majority at screening visit, whichever is greater
ASCVD (including CHD^a^, documented PAD or previous ischaemic stroke) and/or ≥1 additional cardiovascular risk factor^b^
Stable anti-hyperglycaemic treatment (including insulin)
Stable, maximally tolerated dose/regimen of statin for at least 4 weeks prior to screening without other lipid-lowering therapy
Non-HDL-C ≥100 mg/dl (2.59 mmol/l)
TG ≥150 and <500 mg/dl (≥1.70 and <5.65 mmol/l)
No weight variation >5 kg within 3 months
Exclusion criteria
HbA_1c_ ≥9%
Use of any lipid-lowering therapy (other than statin) or over-the-counter product/nutraceuticals known to impact lipids within 4 weeks prior to screening
BMI >45 kg/m^2^
Alcohol consumption >two standard alcoholic drinks/day

*ASCVD* atherosclerotic cardiovascular disease, *BMI* body mass index, *CHD* coronary heart disease, *CKD* chronic kidney disease, *HbA*_*1c*_ glycated haemoglobin, *MI* myocardial infarction, *non*-*HDL*-*C* non-high-density lipoprotein cholesterol, *PAD* peripheral arterial disease, *TG* triglyceride, *UA* unstable angina

^a^History of CHD: acute MI, silent MI, UA, coronary revascularisation procedure or clinically significant CHD diagnosed by invasive or non-invasive testing

^b^Cardiovascular risk factors: hypertension, current smoker, aged ≥45 years (men) and ≥55 years (women), history of micro/macroalbuminuria or diabetic retinopathy, family history of premature CHD, low HDL-C, documented CKD

This trial enrolled adults with T2DM and mixed dyslipidaemia (defined as non-HDL-C ≥100 mg/dl [2.59 mmol/l], and TG ≥150 mg/dl [1.70 mmol/l] and <500 mg/dl [5.65 mmol/l] at the screening visit) that was not adequately controlled with stable maximally tolerated statin therapy for ≥4 weeks prior to the screening visit without other lipid-lowering therapies. Individuals were required to have documented history of atherosclerotic cardiovascular disease (defined as established coronary heart disease, peripheral arterial disease or ischaemic stroke), or at least one additional cardiovascular risk factor in individuals without atherosclerotic cardiovascular disease. The maximally tolerated dose of statin was defined as the highest registered dose/regimen tolerated by the individual based on the investigator’s judgment. Individuals with statin intolerance (as judged by the investigator) documented in medical history, who as a result are no longer on statin therapy, were also eligible to enrol in this study.

Study participants will continue on a stable cholesterol-lowering diet throughout the study, and must be on stable anti-hyperglycaemic therapy (including non-insulin anti-hyperglycaemic agents and insulin) for ≥3 months prior to the screening visit and during the study; changes to anti-hyperglycaemic therapy are allowed only if clinically needed.

Individuals were excluded if they were on any non-statin lipid-lowering therapies (including any over-the-counter products/nutraceuticals known to impact lipids) within 4 weeks prior to the screening visit or during the screening period, had body mass index >45 kg/m^2^ or had glycated haemoglobin (HbA_1c_) ≥9% at the screening visit.

### Study procedures

Individuals were assessed for eligibility during a screening period of up to 3 weeks, followed by randomisation to alirocumab or usual care for a 24-week treatment period. After completing the treatment period, study participants entered a safety observation period of 8 weeks (Fig. 1). On-site clinical assessments are scheduled at weeks—3 (screening), 0 (randomisation), 8, 12, 20 and 24 (end-of-treatment visit), with additional phone visits at weeks 4 and 32.

Eligible individuals were randomised 2:1 to open-label treatment with alirocumab or usual care. At randomisation, treatment was allocated using a centralised treatment allocation system (interactive voice- or web-response system, depending on the study site preference). To ensure balanced treatment groups, randomisation was stratified by the investigator’s choice of usual care therapy, which was pre-specified prior to randomisation.

Usual care includes the option to continue on maximally tolerated statin therapy without the addition of another lipid-lowering therapy at randomisation, or with the addition of one of the following lipid-lowering treatments at randomisation (based on the investigator’s pre-specified choice for that individual participant): ezetimibe, fenofibrate, omega-3 fatty acids or nicotinic acid. The pharmaceutical form, dose and number of units per administration, as well as the timing of dosing of the usual care treatment, will be determined by the investigator and prescribed as per the investigator’s usual practice in accordance with local standard-of-care. The only fibrate that is allowed in this study is fenofibrate. Usual care treatment should be started no later than 7 days from the day of randomisation and should continue for the entire 24-week treatment period. The treatment strategy implemented post randomisation will remain unchanged throughout the study.

For individuals randomised to receive alirocumab treatment, alirocumab is administered subcutaneously with a starting dose of 75 mg every 2 weeks (Q2W) for 24 weeks, with blinded dose increase to 150 mg Q2W at week 12 if non-HDL-C is ≥100 mg/dl (2.59 mmol/l) at week 8. Individuals who have non-HDL-C <100 mg/dl (2.59 mmol/l) at week 8 will continue with alirocumab 75 mg Q2W until the end of the treatment period. All study participants will continue to receive maximally tolerated statin therapy (or no statin if statin-intolerant).

All lipid results collected after randomisation are masked and, unless required for safety reasons, investigators and study participants should not independently evaluate lipid values during the course of the study. For safety reasons, investigators will be informed of TG levels ≥500 mg/dl (5.65 mmol/l).

### Endpoints and assessments

The primary objective of this trial is to demonstrate the superiority of alirocumab in comparison with usual care in the reduction of non-HDL-C after 24 weeks of treatment.

Primary and key secondary endpoints are summarised in Table 2.

**Table 2:** Primary and key secondary endpoints

Primary endpoint
Change (%) in non-HDL-C from baseline to week 24 in ITT population
Key secondary efficacy endpoints
Change (%) from baseline in ITT population
Measured LDL-C at week 24
Non-HDL-C at week 12
Measured LDL-C at week 12
Apo B at week 24
TC at week 24
Lp(a) at week 24
TGs at week 24
HDL-C at week 24
LDL-P number at week 24
Other efficacy endpoints
Change (%) from baseline in ITT population
Calculated LDL-C at weeks 12 and 24
Apo B, TC, Lp(a), HDL-C, TG, and LDL particle number at week 12
Apo A-1, Apo C-III, TRL, LDL-P size, VLDL, HDL and IDL particle number at weeks 12 and 24
Measured LDL-C and TG according to baseline TG (<median or >median) at weeks 12 and 24
Patients (%) reaching
Measured LDL-C <50, 70 and 100 mg/dl at weeks 12 and 24
Non-HDL-C <80, 100 and 130 mg/dl at weeks 12 and 24
≥50% reduction from baseline in measured LDL-C at weeks 12 and 24
Apo B <80 mg/dl at weeks 12 and 24
Absolute change from baseline in Apo B/Apo A-1, TC/HDL-C and LDL-C/HDL-C ratios at weeks 12 and 24
Diabetes-related endpoints
Absolute change from baseline to weeks 12 and 24 in ITT population
HbA_1c_
FPG
Number of glucose-lowering agents
Safety endpoints
TEAEs
AESIs
Product complaints
Laboratory data (including microalbuminuria)
Vital signs (including change in body weight and BMI)
Questionnaire
Treatment acceptance (I-TAQ) at weeks 8 and 24 (for alirocumab arm only^a^)
Other endpoints
Total and free PCSK9 levels at baseline, weeks 12 and 24
Anti-alirocumab antibodies

*AESI* adverse event of special interest, *Apo* apolipoprotein, *BMI* body mass index, *FPG* fasting plasma glucose, *HbA*_*1c*_ glycated haemoglobin, *HDL*-*C* high-density lipoprotein cholesterol, *IDL* intermediate-density lipoprotein, *I*-*TAQ* injection-treatment acceptance questionnaire, *ITT* intent-to-treat, *LDL*-*C* low-density lipoprotein cholesterol, *LDL*-*P*, low-density lipoprotein particle, *Lp(a)* lipoprotein (a), *PCSK9* proprotein convertase subtilisin/kexin type 9, *TC* total cholesterol, *TEAE* treatment-emergent adverse event, *TG* triglyceride, *TRL* triglyceride-rich lipoprotein, *VLDL* very low-density lipoprotein

^a^All individuals randomised to alirocumab who self-injected and filled in the questionnaire at least once

The primary efficacy endpoint is the percent change in non-HDL-C from baseline to week 24, using all values regardless of adherence (intent-to-treat [ITT] approach). Secondary/other efficacy endpoints include the effect of alirocumab versus usual care at weeks 12 and/or 24 on other lipid parameters and sub-fractions (including LDL particle [LDL-P] size and number; and VLDL, HDL and IDL particle number). DM-related endpoints include absolute change from baseline to weeks 12 and 24 in HbA_1c_, fasting plasma glucose (FPG), and the number of glucose-lowering agents. Other endpoints include safety and tolerability (assessed throughout the study for up to 32 weeks), an injection-treatment acceptance questionnaire (I-TAQ) at weeks 8 and 24, changes in plasma PCSK9 levels (assessed at weeks 12 and 24), and development of anti-alirocumab antibodies (assessed at weeks 12 and 24).

Blood sampling to determine all lipid parameters, HbA_1c_ and FPG will be performed in the morning, in fasting condition (for at least 10–12 h and refrain from smoking). Alcohol consumption within 48 h and intense physical exercise within 24 h preceding the blood sampling are discouraged.

TC, HDL-C and TG levels will be directly measured from blood samples by the central laboratory at all clinic visits. Non-HDL-C will be calculated by subtracting HDL-C from TC. LDL-C will be both determined (via beta quantification) by the central laboratory and calculated (using the Friedewald formula) at all clinic visits. TRL will be calculated by TC minus HDL-C minus LDL-C. Apo B, Apo A-1, Apo C-III and Lp(a) will be directly measured by the central laboratory at all visits (except weeks 4 and 32). The ratio of Apo B/Apo A-1 will be calculated, and lipid sub–fractions (LDL-P size and number; and LDL, VLDL, HDL and IDL particle number) will be measured by nuclear magnetic resonance spectroscopy at all visits (except weeks 4 and 32). HbA_1c_ levels will be measured at weeks 0, 12 and 24, and FPG levels will be measured at weeks 0, 8, 12 and 24.

Treatment acceptance will be assessed for study participants randomised to alirocumab at weeks 8 and 24 using the I-TAQ [34], a 22-item validated patient-reported outcome measure. Specifically, the I-TAQ assesses four domains of treatment acceptance: perceived efficacy (i.e. the patient’s perception of whether their treatment is working), acceptance of side effects, injection self-efficacy (i.e. the ability of the patient to perform the task asked of them, e.g. self-injection) and injection convenience. The I-TAQ also has three summary questions measuring overall acceptance.

Safety and tolerability of alirocumab will be assessed throughout the study for up to 32 weeks in terms of treatment-emergent adverse events (TEAEs), adverse events of special interest (described in the Additional file 1: Appendix S3), laboratory parameters, and vital signs.

In a designated secondary analysis, all efficacy endpoints will also be analysed to evaluate the superiority of alirocumab versus usual care in patients who were intended to receive fenofibrate.

### Statistical design and analysis

#### Sample size determination

A total sample size of 420 individuals (280 alirocumab; 140 usual care) was calculated to provide sufficient power for both the primary analysis and the secondary comparison with fenofibrate.

For the primary analysis (alirocumab versus usual care), a total sample size of 219 individuals (146 alirocumab; 73 usual care) is required to achieve 90% power to detect a difference in mean percent change in non-HDL-C between baseline and week 24 at the 0.025 two-sided significance level, assuming that the percent change will be 39% (standard deviation [SD] 28%) and 24% (SD 30%) in the alirocumab and the usual care group, respectively.

For the secondary analysis (alirocumab versus fenofibrate), the required total sample size is 42 individuals (28 alirocumab; 14 fenofibrate) to achieve 90% power to detect a difference in mean percent change in non-HDL-C between baseline and week 24 at the 0.025 two-sided significance level, assuming that the percent change will be 7% (SD 25%) in the fenofibrate subgroup [35]. The total required sample size of 420 individuals was based on the assumption that 10% of study participants will receive fenofibrate therapy, derived from investigator clinical experience provided during study design development.

#### Primary analysis

The primary efficacy analysis population is the ITT population, defined as all randomised individuals with an available baseline non-HDL-C value and at least one available non-HDL-C value within one of the analysis windows up to week 24.

The percent change from baseline to week 24 in non-HDL-C levels will be analysed using a mixed-effect model with repeated measures (MMRM) approach to account for missing data. All available post-baseline data within the analysis window (weeks 8–24) will be used. The MMRM model will include fixed categorical effects of treatment group, time point, randomisation strata, treatment-by-time point interaction, and strata-by-time point interaction as well as the continuous fixed covariates of baseline non-HDL-C value and baseline value-by-time point interaction. Alirocumab will be compared with usual care using appropriate contrasts, and the 97.5% confidence interval of the difference will be provided.

#### Secondary analyses

A hierarchical procedure (concerning key secondary endpoints only) will be used to control the type I error and handle multiple endpoints. If the primary endpoint analysis is significant at an alpha level of 2.5%, secondary endpoints will be tested sequentially using the order defined in Table 2.

Continuous secondary endpoints anticipated to have a normal distribution will be analysed using the same MMRM model as for the primary endpoint with the continuous fixed covariates of corresponding baseline value and baseline value-by-time point interaction. Continuous secondary endpoints anticipated to have a non-normal distribution will be analysed using a multiple imputation approach for handling of missing values, followed by robust regression. Binary secondary endpoints will be analysed using multiple imputation approach for handling of missing values followed by logistic regression.

For the analysis of alirocumab versus fenofibrate, significance will be claimed at the 2.5% alpha level, and the hierarchical procedure will also be used. The comparison of alirocumab versus the other individual options of the usual care will be performed via exploratory subgroup analyses.

Safety analysis will be descriptive, based on the safety population (i.e. all randomised individuals who receive at least one dose or part of a dose of study treatment). Study participants will be analysed according to the treatment actually received (usual care or alirocumab). The safety analysis will focus on the TEAE period, defined as the time from the first open-label dose to the last open-label dose of alirocumab + 70 days (10 weeks) (if randomised to alirocumab) or, if randomised to usual care, 70 days after the last usual care treatment investigational medicinal product has been administered or study day 225, whichever comes first. If the study participant is randomised to usual care and the investigator has not prescribed an additional lipid-lowering therapy, the date of the “last dose of study drug” is defined as the date of the last on-site study visit. Descriptive analyses will be performed for I-TAQ, DM-related endpoints and other endpoints such as anti-alirocumab antibodies.

## Results

Recruitment of study participants was completed in September 2016 with a final randomised population of 413 individuals from 110 active sites in 14 countries (Additional file 1: Appendix S1). For the primary analysis (alirocumab vs usual care), 413 individuals were randomised (276 alirocumab; 137 usual care); approximately 18% of randomised individuals were statin-intolerant. For the analysis of alirocumab versus fenofibrate, 72 individuals were randomised (48 alirocumab; 24 fenofibrate). Since more study participants received fenofibrate than anticipated, recruitment of study participants was stopped earlier than scheduled, as the required sample size to ensure adequate statistical power had been reached.

Main study results of this clinical trial with a 24-week treatment period are expected in the second quarter of 2017.

Full details of baseline characteristics, study population disposition and efficacy and safety results will be available in the future.

## Discussion

The ODYSSEY DM-DYSLIPIDEMIA trial has been designed to investigate the superiority of alirocumab versus usual care in improving non-HDL-C and other lipid parameters in individuals with T2DM and mixed dyslipidaemia at high cardiovascular risk. This is the first trial in the ODYSSEY programme to evaluate non-HDL-C as a primary efficacy endpoint. Findings will supplement available data from the Phase 3 ODYSSEY clinical development programme, which studied non-HDL-C as a secondary efficacy endpoint. Results will also expand on the subgroup analysis in five Phase 3 ODYSSEY placebo-controlled trials of individuals with DM and mixed dyslipidaemia which showed substantial improvements in LDL-C and non-HDL-C levels with alirocumab that were similar to those in individuals with DM without mixed dyslipidaemia [33].

As discussed previously, non-HDL-C levels are believed to provide a better risk estimation of cardiovascular disease than LDL-C, especially in individuals with DM and mixed dyslipidaemia, and statins are commonly used to modulate non-HDL-C levels. However, despite statin treatment, many individuals with DM and dyslipidaemia remain sub-optimally treated, and many go on to experience cardiovascular events [22]. A second lipid-lowering therapy is frequently used to bring non-HDL-C to within the target range. Currently, there is no single preferred second-line treatment, and guidelines typically suggest an individualised approach [4, 9–12]. The ODYSSEY DM-DYSLIPIDEMIA study, comparing alirocumab with usual care (which includes other lipid-lowering therapies currently used to manage mixed dyslipidaemia, namely ezetimibe, fibrates, nicotinic acid and omega-3 fatty acids), allows physicians participating in the study to make an individualised therapeutic choice for each study participant whose non-HDL-C remains elevated despite maximally tolerated statin therapy.

In addition to individuals on maximally tolerated statin therapy, this study also assessed the efficacy and safety of alirocumab in individuals with DM and mixed dyslipidaemia who are statin-intolerant or are on alternate-day dosing regimens of statins. Furthermore, this study is also powered to assess the superiority of alirocumab versus fenofibrate. Fenofibrate therapy, which has not definitively demonstrated cardiovascular outcome benefits in a randomised trial setting, has been suggested to reduce cardiovascular risk when co-administered with a statin in individuals with DM with elevated TG and low HDL-C [16, 17, 36].

Distinct from previous studies in the ODYSSEY Phase 3 clinical development programme, this trial will also measure LDL-P number and size, and Apo C-III. Individuals with T2DM are known to have a higher proportion of atherogenic, small, dense LDL particles than those without DM. These small, dense LDL-P, which are more susceptible to glycation or oxidation, decrease their affinity for the LDL receptor (LDLR) and contribute to reduced LDL catabolism [2]. In a post hoc substudy of a Phase 2 trial, alirocumab has been shown to significantly reduce both LDL-C and LDL-P concentrations in hypercholesterolaemic individuals without DM on stable statin therapy [37]. Apo C-III, an important regulator of TG levels, has been shown to be an independent cardiovascular risk factor in individuals with DM [38]. Apo C-III genetic deficiencies are associated with low TG levels and reduced risk of coronary heart disease [39]. In a post hoc analysis of three Phase 2 trials, alirocumab was shown to reduce Apo C-III levels by 14.5–19.1% in non-diabetic individuals with LDL-C ≥100 mg/dl (2.59 mmol/l) on stable statin therapy [40]. This effect may be a result of increased clearance or reduced production of VLDL particles rather than an impact on Apo C-III synthesis. Interestingly, recent evidence suggested that Apo C-III might inhibit clearance of TRL through a mechanism closely related to LDL family receptors (i.e. LDLR and LDLR–related protein 1) [41]; whether alirocumab and PCSK9 inhibitors impact this mechanism may be an area of future research. Furthermore, it may be hypothesised that increased hepatic PCSK9 expression is involved in the initiation of mixed dyslipidaemia [42–44]. However, the blocking of the PCSK9 extracellular pathway with PCSK9 monoclonal antibodies does not clinically meaningfully reduce plasma TG levels in the Phase 3 trials, though notably they were mainly conducted in normotriglyceridaemic subjects [45]. As statins have a greater TG-lowering effect in those with higher baseline TG levels [17], it is of interest that this study will evaluate the effect of PCSK9 inhibition in patients with elevated levels of TGs at baseline.

There is currently high interest as to whether PCSK9 inhibitors affect glucose homeostasis following recent reports suggesting a potential link between PCSK9 and DM [46–48]. A previous pooled analysis of 10 Phase 3 ODYSSEY clinical trials including ~5000 individuals (following 24–104 weeks of follow-up) did not find evidence of changes in glycaemic control following treatment with alirocumab [32]. To expand on these findings, the ODYSSEY DM-DYSLIPIDEMIA study will capture changes to DM-related endpoints including HbA_1c_, FPG, and the number of glucose-lowering agents. Further valuable information on the use of alirocumab in individuals with DM will be provided by the ongoing ODYSSEY DM-INSULIN trial, which investigates alirocumab in individuals with T1 and T2DM at high cardiovascular risk who are being treated with insulin [49]. Moreover, the recent discontinuation of the clinical development of bococizumab, due to loss of efficacy over time and immunogenicity issues [25, 26], has also sparked interest in the development and effect of anti-drug antibodies. A pooled analysis of 10 ODYSSEY trials showed very low rates of immunogenicity with alirocumab, without loss of efficacy over time [27]. Anti-alirocumab antibodies will be measured throughout the study to further assess immunogenicity in this specific population of individuals with T2DM and mixed dyslipidaemia.

DM-DYSLIPIDEMIA also explores treatment acceptance through the I-TAQ, a patient-reported outcome measure [34]. Alirocumab requires patients to self-administer the medication via subcutaneous injections, a treatment strategy that has previously been rarely used in the management of hyperlipidaemia; therefore, understanding patients’ perspectives about using injection treatments versus oral medications is important but difficult to measure with existing methods. Based on previous data, the majority of study participants and physicians have considered the alirocumab pre-filled pen to be easy to use, and participants have shown a willingness to self-inject [50]; high rates of treatment adherence (~98%) have been reported with alirocumab [34]. Further information on treatment acceptance will be provided by DM-INSULIN, which investigates alirocumab in individuals being treated with insulin (and hence already familiar with self-injection) [49].

## Conclusions

Together with the ODYSSEY DM-INSULIN study, the ODYSSEY DM–DYSLIPIDEMIA trial will provide valuable information on the efficacy and safety of alirocumab in individuals with DM. The large ongoing ODYSSEY OUTCOMES trial (NCT01663402), which randomised patients within 1 year post acute coronary syndrome and includes a significant number of patients with DM, will provide data on the benefit of alirocumab on cardiovascular morbidity and mortality. These trials may ultimately help guide clinical decision-making beyond statin therapy in this high-risk population.

## Additional file

**Additional file 1:** Appendices.

## Abbreviations

Apo: apolipoprotein
DM: diabetes mellitus
FPG: fasting plasma glucose
HbA_1c_: glycated haemoglobin
HDL-C: high-density lipoprotein cholesterol
IDL: intermediate-density lipoprotein
I-TAQ: injection-treatment acceptance questionnaire
ITT: intent-to-treat
LDL: low-density lipoprotein
LDL-C: low-density lipoprotein cholesterol
LDL-P: low-density lipoprotein particle
LDLR: low-density lipoprotein receptor
Lp(a): lipoprotein(a)
MMRM: mixed effect model with repeated measures
non-HDL-C: non-high-density lipoprotein cholesterol
PCSK9: proprotein convertase subtilisin/kexin type 9
Q2W: every 2 weeks
SD: standard deviation
T2DM: type 2 diabetes mellitus
TC: total cholesterol
TEAE: treatment-emergent adverse events
TG: triglyceride
TRL: triglyceride-rich lipoprotein
VLDL: very low-density lipoprotein
