# Supplementary material for: Design and rationale of the ODYSSEY DM-DYSLIPIDEMIA trial: lipid-lowering efficacy and safety of alirocumab in individuals with type 2 diabetes and mixed dyslipidaemia at high cardiovascular risk
Source: Cardiovasc Diabetol. 2017 May 25;16:70. doi: 10.1186/s12933-017-0552-4 (PMC5445362; doi:10.1186/s12933-017-0552-4)
Supplement: Supplementary file 1 — Additional file 1: Appendices. [file 12933_2017_552_MOESM1_ESM.docx]

# Additional file 1: Appendices

**Appendix S1. Study investigators and committee members**

The ODYSSEY DM-DYSLIPIDEMIA is a multicentre study being conducted at 110 active sites in 14 countries. Below is the list of principal investigators who have screened at least one patient (national coordinators are in bold, where applicable). Of these, no patients were randomised in Kuwait.

**Australia:** Gregory Fulcher (Sydney), Michael d'Emden (Herston), Claire Morbey (Merewether).

**Brazil:** Francisco Fonseca (Sao Paulo), Gustavo Augusto (Sao Paulo), Adriana Forti (Fortaleza), Jose Francisco Saraiva (Campinas), João Salles (Sao Paulo).

**Finland: Olavi Ukkola (Oulu)**, Jorma Strand (Oulu), Jyrki Taurio (Tampere).

**Germany: Andreas Birkenfeld (Dresden)**, Thomas Schaum (Oldenburg in Holstein), Christiane Klein (Künzing), Petra Ott (Dippoldiswalde), Julia Chevts (Karlsruhe), Ulrich Weber (Essen), Axel Schaefer (Essen), Thorsten Krause (Goch), Kristof Graf (Berlin).

**Kuwait: Abdullah Bennakhi (Kuwait)**.

**Israel:** Ilana Herman (Beer-Sheva), Hilla Knobler (Rehovot), Dror Dicker (Petach Tikva), Ilan Shimon (Petach Tikva), Naftali Stern (Tel-Aviv).

**Italy: Stefano Del Prato (Pisa)**, Ezio Ghigo (Torino), Angela Rivellese (Napoli), Roberto Trevisan (Bergamo), Andrea Giaccari (Rome), Vincenzo Provenzano (Partinico), Agostino Gnasso (Catanzaro), Alberto Zambon (Padova).

**Lebanon: Sami Azar (Beirut)**, Marie Merheb (Beirut).

**Norway: Gisle Langslet (Oslo)**, Eli Heggen (Oslo).

**Sweden: Mats Eriksson** (Stockholm), Stefano Romeo (Göteborg).

**Switzerland: Andre Miserez (Reinach)**, Gottfried Rudofsky (Olten).

**Turkey: Fusun Saygili** (Izmir), Tevfik Demir (Izmir), Emre Bozkirli (Adana), Fahri Bayram (Kayseri), Kerim Kucukler (Corum), Cumali Gokce (Hatay).

**United Arab Emirates: Khadija Hafidh (Dubai)**.

**United Kingdom: Adie Viljoen (Stevenage)**, Jamie Smith (Torquay), Elizabeth Hughes (West Bromwich), Bijay Vaidya (Devon), Arutchelvam Vijayaramen (Middlesbrough), Handreen Soran (Manchester).

**United States:** Atoya Adams (Las Vegas, NV), Samir Arora (Columbus, OH), Vivek Awasty (Marion, OH), Masoud Azizad (Northridge, CA), Seth Baum (Boca Raton, FL), Harold Bays (Louisville, KY), Eric Bolster (Summerville, SC), Christopher Case (Jefferson City, MO), Louis Chaykin (Bradenton, FL), George Dailey (La Jolla, CA), Juan Frias (Los Angeles, CA), Anne Goldberg (Saint Louis, MO), Yehuda Handelsman (Tarzana, CA), Cynthia Huffman (Tampa, FL), James LaRocque (Chesapeake , VA), Steven Leichter (Columbus, GA), Michael Lillestol (Fargo, ND), Kathyrn Lucas (Morehead City, NC), Jose Mandry (Ocoee, FL), Mustafa Mandviwala (Tomball, TX), Travis Monchamp (Bend, OR), Jon McRae (Bainbridge, GA), Frank Mikell (Springfield, IL), Samer Nakhle (Las Vegas, NV), Paul Norwood (Fresno, CA), Kerem Ozer (Round Rock, TX), Rakesh Patel (Houston, TX), Michael Reeves (Chattanooga, TN), Marc Rendell (Omaha, NE), Jackson Rhudy (Salt Lake City, UT), Helena Rodbard (Rockville, MD), Paul Rosenblit (Huntington Beach, CA), Julio Rosenstock (Dallas, TX), Jean-Louis Selam (Tustin, CA), Stephen Smith (Morganton, NC), William Smith (Knoxville, TN), Dan Streja (West Hills, CA), Danny Sugimoto (Chicago, IL), James Thrasher (Little Rock, AR), Carl Vance (Idaho Falls, ID), Francisco Velazquez (Houston, TX), Ronald Watts (Stockbridge, GA), Debra Weinstein (Boynton Beach, FL), Franklin Zieve (Richmond, VA), Jeffrey Geohas (Evanston, IL), Nizar Daboul (Maumee, OH), Eugene Soroka (Port Hueneme, CA), Bradley Block (Oviedo, FL), Howard Weintraub (New York, NY), Raymond de la Rosa (Fort Smith, AR), Hiralal Maheshwari (Crystal Lake, IL), Charles Dahl (Orem, UT), Philip Nicol (Murrels Inlet, SC), Walter Pharr (Greensboro, NC), Jonathan Wise (Metairie, LA).

**Steering Committee:** Lawrence A. Leiter (Toronto, Canada), Chair; Bertrand Cariou (Nantes, France); Helen M. Colhoun (Edinburgh, UK); Stefano Del Prato (Pisa, Italy); Robert R. Henry (San Diego, CA, USA); Dirk Mϋller-Wieland (Aachen, Germany); Kausik K. Ray (London, UK); Francisco J. Tinahones (Málaga, Spain).

**Appendix S2. Inclusion and exclusion criteria**

## B.1 Full inclusion criteria

- Aged ≥ 18 years or legal age of majority at screening visit, whichever is greater.
- Individuals with type 2 diabetes mellitus and mixed dyslipidaemia not adequately controlled on a stable maximally tolerated dose/regimen of statins for ≥ 4 weeks prior to the screening visit (without other lipid-lowering therapies).
- Non-high-density lipoprotein-cholesterol (non-HDL-C) ≥ 100 mg/dl (2.59 mmol/l).
- Triglycerides (TG) ≥ 150 mg/dl (1.70 mmol/l) and < 500 mg/dl (5.65 mmol/l).
- Documented history of atherosclerotic cardiovascular disease (defined as established coronary heart disease, documented peripheral arterial disease or documented previous ischaemic stroke) or ≥ 1 additional cardiovascular risk factor (see below for definitions).
- On stable anti-hyperglycaemic agents for ≥ 3 months (including stable insulin dose, defined as no variation > 30% in daily insulin dose within the preceding 3 months).
- No variation of weight > 5 kg and on stable dose of medications that are known to influence weight and/or lipids (other than lipid-lowering therapies, such as oestrogen replacement, oral steroids or antipsychotics known to impact weight), within 3 months prior to the screening visit or between screening and randomisation.
- Signed written informed consent was required from all study participants.

The definition of maximally tolerated dose/regimen is the highest registered dose/regimen tolerated by the individual, based on the judgment of the investigator. Individuals not able to be on a maximum dose/regimen of statin should be treated with the dose of statin which is considered appropriate for the individual as per the investigator’s judgment or concerns. Some examples of acceptable reasons for an individual taking a lower statin dose include, but are not limited to: adverse effects on higher doses, advanced age, low body mass index (BMI), regional practices, local prescribing information and concomitant medications. Individuals may be on an alternate‑day dose of statin as long as the dose is consistently taken. Concomitant treatment with more than one statin is not permitted. Individuals who have documented statin intolerance as judged by the investigator and who are no longer on statin therapy as a result will also be eligible for the study. The reason(s) for not being on a maximum dose/regimen of statin (including statin intolerance) will need to be documented in the case report form.

### B.1.1 Atherosclerotic Cardiovascular Disease

#### B.1.1.1 Established Coronary Heart Disease

This is defined as a documented history of coronary heart disease, including one or more of the following: acute myocardial infarction (MI), silent MI, unstable angina (UA), coronary revascularisation procedure (e.g. percutaneous coronary intervention [PCI] or coronary artery bypass graft surgery [CABG]) and clinically significant coronary heart disease diagnosed by invasive or non-invasive testing (such as coronary angiography, stress test using treadmill, stress echocardiography or nuclear imaging).

#### B.1.1.2 Documented peripheral arterial disease

Documented peripheral arterial disease is defined as one of the following criteria:

1. Current intermittent claudication (muscle discomfort in the lower limb that is both reproducible and produced by exercise and relieved by rest within 10 minutes) of presumed atherosclerotic origin *together with* ankle-brachial index ≤ 0.90 in either leg at rest, OR
2. History of intermittent claudication *together with* endovascular procedure or surgical intervention in one or both legs because of atherosclerotic disease, OR
3. History of critical limb ischaemia *together with* thrombolysis, endovascular procedure or surgical intervention in one or both legs because of atherosclerotic disease.

#### B.1.1.3 Documented previous ischaemic stroke

Documented previous ischaemic stroke with a focal ischaemic neurological deficit that persisted for more than 24 hours, considered as being of atherothrombotic origin. Computed tomography or magnetic resonance imaging must have been performed to rule out haemorrhage and non-ischaemic neurological disease.

### B.1.2 Cardiovascular risk factors

Cardiovascular risk factors include at least one of the following:

1. Hypertension (established on antihypertensive medicine).
2. Current cigarette smoker.
3. Age ≥ 45 years for men; ≥ 55 years for women.
4. History of micro/macroalbuminuria.
5. History of diabetic retinopathy (preproliferative or proliferative).
6. Family history of premature coronary heart disease (in father or brother before 55 years of age; in mother or sister before 65 years of age).
7. Low HDL-C (male < 40 mg/dl [1.0 mmol/l] and female < 50 mg/dl [1.3 mmol/l]).
8. Documented chronic kidney disease as defined by 15 ≤ estimated glomerular filtration rate (eGFR) < 60 ml/min/1.73 m^2^ for 3 months or more, including the screening visit.

## B.2 Full exclusion criteria

Individuals who have met all the above inclusion criteria will be screened for the following exclusion criteria.

### B.2.1 Exclusion criteria related to study methodology

- Individuals that are not on a stable dose/regimen of statin for at least 4 weeks prior to the screening visit or from screening to randomisation, unless statin‑intolerant, in which case there will be no statin for 4 weeks prior to the screening visit/during the screening period.
- Use of any lipid-lowering therapies other than statins within 4 weeks prior to the screening visit or during the screening period (e.g. ezetimibe, fenofibrate, nicotinic acid, omega-3 fatty acids, etc.).
- Use of over-the-counter products/nutraceuticals known to impact lipids (e.g. red yeast rice) within 4 weeks prior to the screening visit or during the screening period.
- On insulin at baseline and not treated with insulin for at least 6 months prior to the screening visit and not on a stable insulin regimen (i.e. a change in type of insulin, general timing/frequency of injections, mode or pattern of administration such as basal only, basal-prandial, etc.) for at least 3 months prior to the screening visit, or likelihood of requiring a change in insulin type/frequency or mode of injection during the study period.
- Likelihood of requiring a change of antihyperglycaemic regimen during the course of the study, as judged by the investigator (e.g. addition of new agent, plans for titration of insulin dose, etc.).
- Recent (within 3 months prior to the screening visit or between screening and randomisation visits) MI, UA leading to hospitalisation, uncontrolled cardiac arrhythmia, CABG, PCI, carotid surgery or stenting, cerebrovascular accident, transient ischaemic attack, endovascular procedure or surgical intervention for peripheral vascular disease.
- Planned to undergo scheduled PCI, CABG, or carotid or peripheral revascularisation during the study.
- History of New York Heart Association Class III or IV heart failure within the past 12 months.
- Systolic blood pressure >180 mmHg or diastolic blood pressure > 110 mmHg at screening or randomisation visit.
- Plasmapheresis within 2 months prior to the screening visit, or planned plasmapheresis.
- Known history of haemorrhagic stroke.
- Known history of loss of function of proprotein convertase subtilisin/kexin type 9 (i.e. genetic mutation or sequence variation) or known history of homozygous familial hypercholesterolaemia.
- New cancer or active progression of cancer within the past 5 years, except for adequately treated basal cell skin cancer, squamous cell skin cancer or in situ cervical cancer.
- Known history of positive HIV test.
- Use of any active investigational drugs within 1 month or five half-lives, whichever is longer.
- Individuals not previously instructed on a cholesterol-lowering diet prior to the screening visit.
- BMI > 45 kg/m^2^ at screening or currently enrolled in a weight loss program and still in active phase of weight loss, as judged by investigator; recent initiation of weight loss drugs (within 3 months prior to the screening visit) or recent bariatric surgery (within the last 6 months) and in an active weight loss phase, as judged by the investigator, or plans to undergo bariatric surgery or to initiate weight loss drugs during the course of the study.
- Drinking > 2 standard alcoholic drinks per day (one standard drink is considered as one pint/bottle of beer, one glass of wine, or one shot of hard liquour).
- Receiving or plans to receive renal replacement therapy during the study (e.g. haemodialysis, renal transplant).
- Presence of any clinically significant uncontrolled endocrine disease known to influence serum lipids or lipoproteins (note: individuals on thyroid replacement therapy can be included if the dosage of thyroxin has been stable for ≥ 3 months prior to screening and the individual’s sensitive thyroid-stimulating hormone levels are within ±10% of the normal range of the laboratory at the screening visit).
- Known familial hypertriglyceridaemia (Fredrickson classification 1 and 5).
- Laboratory findings during the screening period (not including randomisation assessments, except for pregnancy test):
  - Serum TGs > 500 mg/dl (5.65 mmol/l) (one repeat assessment is allowed).
  - Positive serum or urine pregnancy test in women of childbearing potential.
  - Positive test for hepatitis B surface antigen or hepatitis C antibody (confirmed by reflexive testing).
  - eGFR < 15 ml/min/1.73 m^2^ according to 4-variable Modification of Diet in Renal Disease equation.
  - Alanine aminotransferase (ALT) or aspartate aminotransferase (AST) > 3 x upper limit of normal range (ULN) (one repeat assessment is allowed).
  - Creatine phosphokinase > 3 x ULN (one repeat assessment is allowed).
  - Glycated haemoglobin ≥ 9%.
- Conditions/situations such as:
  - Individuals with short life expectancy.
  - Requirement for concomitant treatment that could bias primary evaluation.
  - Impossibility to meet specific protocol requirements (e.g. need for hospitalisation, ability to make study visits).
  - Investigator or any sub-investigator, research assistant, pharmacist, study coordinator, other staff, or relative thereof directly involved in the conduct of the study.
  - Individual is uncooperative or any condition that could make the individual potentially non-compliant to the study procedures.
  - Any technical/administrative reason that makes it impossible to randomise the individual in the study.
  - Any clinically significant abnormality identified at the time of screening that, in the judgment of the investigator or any sub-investigator, would preclude safe completion of the study or constrain endpoints assessment such as major systemic diseases (e.g. history of acute pancreatitis).

### B.2.2 Exclusion criteria related to background therapy

Any contraindications to the background therapies or warning/precaution of use (when appropriate) as displayed in the respective national product labelling.

### B.2.3 Exclusion criteria related to alirocumab

Known hypersensitivity to alirocumab or to any of the ingredients of alirocumab; pregnant or breastfeeding women; women of childbearing potential not protected by highly effective contraceptive methods of birth control (as defined in the informed consent form and/or in a local protocol addendum) and/or who are unwilling or unable to be tested for pregnancy. Women of childbearing potential must have a confirmed negative pregnancy test at screening and inclusion visits. They must use an effective contraceptive method throughout the entire duration of the study treatment and for at least 10 weeks after the last study drug administration. The applied methods of contraception must meet the criteria for a highly effective method of birth control according to the International Conference on Harmonization of Technical Requirements for Registration of Pharmaceuticals for Human Use. M3(R2): Guidance on nonclinical safety studies for the conduct of human clinical trials and marketing authorisation for pharmaceuticals ICH.2009 Jun: 1-25. Postmenopausal women must be amenorrhoeic for at least 12 months.

### B.2.4 Additional exclusion criteria during or at the end of screening or run-in phase before randomisation

Individuals who have withdrawn consent before enrolment/randomisation (starting from signed informed consent form); stopped enrolment/randomisation in the study, despite screening of the individual.

**Appendix S3. Adverse events of special interest**

Adverse events of special interest include:

- Increase in ALT ≥ 3 x ULN (if baseline ALT < ULN) or ALT ≥ 2 times the baseline value (if baseline ALT ≥ ULN).
- Pregnancy occurring in a female study participant or the partner of a male study participant.
- Symptomatic overdose (accidental or intentional) with alirocumab.
- Allergic events (including local injection-site reactions that are allergic in nature) requiring consultation with another physician.
- Neurologic events requiring additional examinations/procedures and/or consultation with a specialist.
- Neurocognitive events.
